# A novel acridine derivative, LS-1-10 inhibits autophagic degradation and triggers apoptosis in colon cancer cells

**DOI:** 10.1038/cddis.2017.498

**Published:** 2017-10-05

**Authors:** Wan Fu, Xue Li, Xiaopeng Lu, Luyao Zhang, Ran Li, Nan Zhang, Shan Liu, Xin Yang, Yue Wang, Ying Zhao, Xiangbao Meng, Wei-Guo Zhu

**Affiliations:** 1 Key Laboratory of Carcinogenesis and Translational Research (Ministry of Education), State Key Laboratory of Natural and Biomimetic Drugs, Beijing Key Laboratory of Protein Posttranslational Modifications and Cell Function, Department of Biochemistry and Molecular Biology, School of Basic Medical Sciences, Peking University Health Science Center, Beijing 100191, China; 2 Department of Biochemistry and Molecular Biology, School of Medicine, Shenzhen University, Shenzhen 518060, China; 3 Peking-Tsinghua University Center for Life Science, Peking University, Beijing 100871, China

## Abstract

Autophagy promotes cancer cell survival and drug resistance by degrading harmful cellular components and maintaining cellular energy levels. Disruption of autophagy may be a promising approach to sensitize cancer cells to anticancer drugs. The combination of autophagic inhibitors, such as chloroquine (CQ) and lucanthone with conventional cancer therapeutics has been investigated in clinical trials, but adverse drug–drug interactions are a high possibility. Here we designed and synthesized a novel, small-molecule library based on an acridine skeleton and the CQ structure with various modifications and substitutions and screened the compounds for effective autophagy inhibition. We found that 9-chloro-2-(3-(dimethylamino)propyl)pyrrolo[2,3,4-kl]acridin-1(2H)-one (LS-1-10) was the most effective from our library at inhibiting autophagic-mediated degradation and could decrease the viability of multiple colon cancer cells. In addition, LS-1-10 induced DNA damage and caspase 8-mediated apoptosis. Overall, this small molecule was more efficient at reducing the viability of cancer cells than other conventional chemotherapeutic agents, such as CQ and amsacrine. The anticancer and autophagy-inhibiting activities of LS-1-10 were confirmed *in vivo* in a xenograft mouse model. Collectively, this study has identified a new and efficient single compound with both autophagy-inhibiting and anticancer activity, which may provide a novel approach for cancer therapy.

Autophagy is an important catabolic process that is highly conserved across all eukaryotes.^1, 2, 3, 4^ It is a protein degradation pathway by which cytoplasmic constituents are delivered to lysosome for digestion.^5^ This process is induced in response to various stimuli, such as genotoxic chemicals, oxidative reagents and starvation, to maintain cellular metabolism and eliminate harmful damaged proteins and organelles, thus facilitate cell survival.^6, 7^ Numerous studies have identified a complex association between autophagy and cancer development.^8, 9, 10^ Many cancer therapeutics, including DNA damaging agents, histone deacetylase inhibitors and ionizing radiation induce high levels of autophagy to confer cytoprotection of cancer cells.^11, 12, 13, 14, 15^ Inhibition of autophagy enhances the pro-apoptotic effects of anticancer agents and thus may be a promising strategy to augment the activity of many cancer therapeutics.^16^

Many combination therapies are undergoing clinical trials to verify whether adjunctive autophagy inhibitors can enhance the anticancer efficacy of small-molecule drugs.^16, 17^ Chloroquine (CQ), lucanthone, and their analogs, are currently the only autophagic inhibitors under clinical investigation for use as cancer therapeutics.^18, 19, 20^ However, CQ can induce ocular toxicity and irreversible retinopathy,^21^ and clinical trials of lucanthone were prematurely terminated or suspended for yet unknown reasons. Additional inhibitors of autophagy are being developed with the aim of enhancing the activity of chemotherapeutic agents. Adverse drug–drug interactions may arise from these complex drug combinations, thus the development of a small, single molecule that possesses both potent anticancer and anti-autophagy activity is required.

Acridine derivatives, such as amsacrine (m-AMSA) and DACA,^22, 23, 24^ exhibit DNA-intercalating and topoisomerase-inhibiting activity and are prime candidates as anticancer agents.^25^ m-AMSA has been used to treat acute leukemia and malignant lymphoma, but is ineffective against solid tumors.^22, 26, 27, 28, 29^ Acridine provides an ideal scaffold as an anti-tumor drug for two reasons. First, the linear tricyclic aromatic structure of acridine ensures high DNA intercalation. Second, modifications to the chemical structure, such as the side chain on the pyridine ring, can generate numerous biologically active compounds with different activities.^30^

Here, we generated a novel acridine derivative (hereafter known as LS-1-10) that contains a quinoline moiety and a flexible tertiary-amine side chain similar to that of CQ and hydrochloroquine (HCQ). We verified that LS-1-10 acts as a DNA damaging agent and can simultaneously inhibit autophagy. We found that LS-1-10 can reduce the viability of various colon cancer cell lines with a higher efficacy than many conventional chemotherapeutic agents. Taken together, LS-1-10 possesses a dual function as a DNA damaging agent and inhibitor of autophagy. We propose that LS-1-10 may be exploited as a suitable small-molecule drug in colon cancer therapy.

## Results

### Screening acridine derivatives with a similar structure to CQ

**Figure 1:**
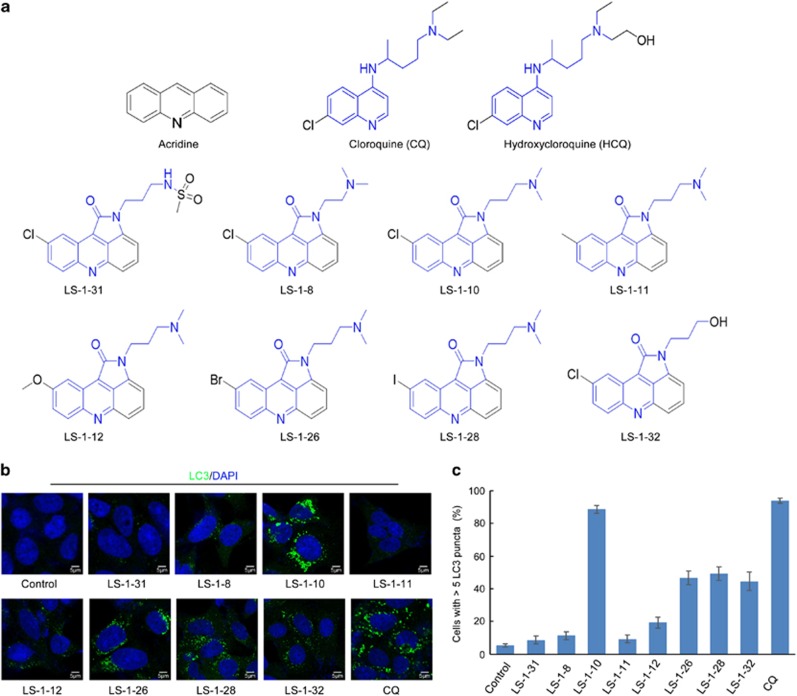
Screen for acridine derivatives that inhibit autophagic degradation. (**a**) Structures of a series of small molecules with an acridine skeleton and structure similar to chloroquine. (**b**) DLD1 cells were treated with 5 *μ*M acridine derivative or 10 *μ*M chloroquine for 24 h. Immunofluorescence was performed after staining with an anti-MAP1LC3 antibody. Scale bars, 5 *μ*m. (**c**) Quantification of the MAP1LC3-positive punctate cells shown in (**b**). Only cells with more than five puncta were counted. Data represent the means±S.D. (*n*=3)

Most DNA damaging agents, including m-AMSA, induce autophagy and thus promote cancer cell survival.^31^ Here, we designed and synthesized a series of small molecules based on the skeleton of acridine and the structures of CQ and HCQ (Figure 1a) with the aim of developing a drug with both anticancer and autophagy-inhibiting functions. Autophagy can be monitored by the accumulation of the autophagy marker LC3 and the degradation of p62.^32^ Inhibition of autophagic degradation usually causes accumulations and puncta formations of both LC3-I/II and p62.^32^ Thus, we analyzed the abundance and distribution of these two biological markers after treating DLD1 and LoVo human colon cancer cell lines with eight in-house generated molecules (Figures 1b and c, S1A). Among the eight molecules tested, three showed potential to effectively inhibit autophagic degradation (Figure 1b, Supplementary Figure S1A):

LS-1-10 [9-chloro-2-(3-(dimethylamino)propyl)pyrrolo[2,3,4-kl]acridin-1(2H)-one]

LS-1-26 [9-bromo-2-(3-(dimethylamino)propyl)pyrrolo[2,3,4-kl]acridin-1(2H)-one]

LS-1-28 [2-(3-(dimethylamino)propyl)-9-iodopyrrolo[2,3,4-kl]acridin-1(2H)-one].

Among the three compounds, LS-1-10 was the most effective at inducing LC3 puncta accumulation and cleavage of PARP-1, a marker of apoptosis (Figures 1b and c,Supplementary Figure S1A). We considered, therefore, that LS-1-10 would have the highest potential as an anticancer drug, and thus focused our subsequent analyses on this compound.

### LS-1-10 inhibits autophagic degradation

**Figure 2:**
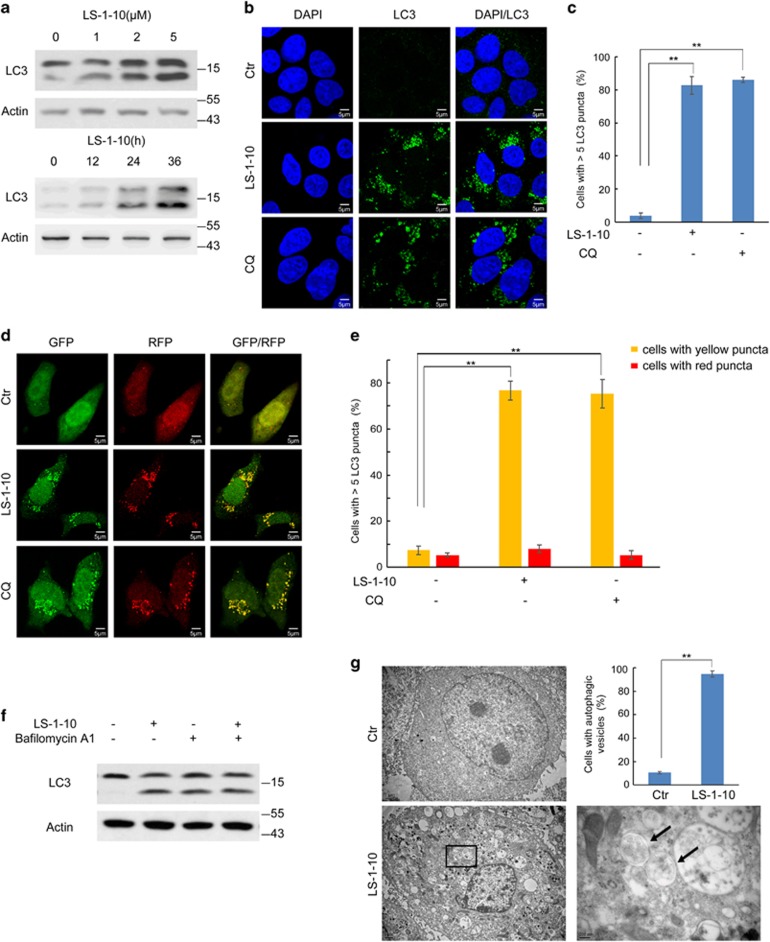
LS-1-10 inhibits autophagic degradation. (**a**) DLD1 cells were treated with different concentrations of LS-1-10 (1, 2 and 5 *μ*M) for 24 h (upper panel) or 5 *μ*M LS-1-10 over a time course (lower panel). Immunoblotting was performed using an LC3 antibody. (**b**) DLD1 cells were incubated with 5 *μ*M LS-1-10 or 10 *μ*M chloroquine (CQ) for 24 h. Immunofluorescence was performed after staining with an LC3 antibody. Scale bars, 5 *μ*m. (**c**) Quantification of the LC3-positive punctate cells shown in (**b**). Only cells with more than five puncta were counted. Data represent the means±S.D. (*n*=3). Student’s *t*-test, **P<0.01. (**d**) DLD1 cells were transfected with ptfLC3 and then treated with 5 *μ*M LS-1-10 or 10 *μ*M CQ for 24 h. The distribution of GFP/RFP-LC3 was examined by confocal microscopy. (**e**) Quantification of the LC3 puncta shown in (**d**). Data represent the means±S.D. based (*n*=3). Student’s *t*-test, **P<0.01. (**f**) DLD1 cells were treated with 5 *μ*M LS-1-10, 100 nM bafilomycin A1, or both for 24 h. Whole-cell lysates were extracted for immunoblotting with the indicated antibodies. (**g**) Autophagic vesicles or autophagosomes in DLD1 cells were observed by electron microscopy after 24 h of 5 *μ*M LS-1-10 treatment. Arrows in the enlargement indicate autophagic vesicles. The graph shows a statistical analysis of autophagic vesicles in DLD1 cells upon LS-1-10 treatment. Data represent the means±S.D. (*n*=3). Student’s *t*-test, **P<0.01

To confirm how effectively LS-1-10 can inhibit autophagic degradation, we monitored the accumulation and puncta formation of endogenous LC3 in DLD1 cells treated with LS-1-10 or CQ as a positive control.^32^ LS-1-10 induced the accumulation of LC3-I/II in a time-dependent and dose-dependent manner (Figure 2a) and the formation of large numbers of LC3 puncta (Figures 2b and c). Some compounds, such as 3-methyladenine (3-MA), elicit opposing effects on autophagy depending on the dosage or timing of treatment. We therefore evaluated the consequences of treating cells with a high dose (20 *μ*M) of LS-1-10 and obtained similar results to those in our original dose–response curve (Supplementary Figure S2A). LS-1-10 treatment also resulted in LC3-I/II accumulation in other human cancer cell lines, including HT-29, SW480, A549 and U2OS cells (Supplementary Figure S2B). These data may indicate a disruption of autophagic degradation in these cells.

Contrary to LC3 accumulation, we detected a reduction in p62 protein levels following cellular exposure to LS-1-10 (Supplementary Figure S2D). However, p62 mRNA levels were upregulated (Supplementary Figure S2C). p62 is a shuttle protein that transports poly-ubiquitinated proteins for proteasomal and autophagy-dependent degradation.^33, 34, 35, 36^ We evaluated whether the reduction in p62 protein level was mediated by autophagy. To this aim, we generated an autophagy-deficient DLD1 cell line (ATG3KO) by CRISPR/Cas9 deletion of ATG3 (Supplementary Figure S2D) and found that p62 expression decreased upon LS-1-10 treatment in both wild-type (WT) and ATG3KO cells. These data suggest that the reduction in p62 protein is autophagy-independent (Supplementary Figure S2C). Furthermore, immunostaining showed that p62 co-localized with LC3 puncta, indicating that its autophagic degradation was blocked upon LS-1-10 treatment (Supplementary Figure S2E). Thus, we conclude that p62 undergoes autophagy-independent degradation upon LS-1-10 treatment, and is not a suitable marker to study autophagy in our system.

One of the limitations of the above experiments is that they fail to distinguish whether the augmentation of autophagosome markers is due to enhanced autophagosome biogenesis or decreased clearance.^32^ To discriminate between these two possibilities, we used a ptfLC3 expression vector encoding LC3 fused to monomeric red fluorescent protein (RFP) and green fluorescent protein (GFP) in tandem.^37^ Red and green fluorescence from this construct is visible in autophagosomes. In autolysosomes, the GFP signal is weak due to its labile expression in acid conditions, whereas the RFP signal is stable and yields persistent red fluorescence.^37^ We transfected DLD1 cells with the ptfLC3 vector and exposed them to LS-1-10 or CQ. After treatment, the cells manifested a high density of puncta that were visible in both the red and green fluorescence channels, which suggested disruption of autophagic degradation (Figures 2d and e). To further verify the autophagic flux, we treated DLD1 cells with both LS-1-10 and bafilomycin A1 – an inhibitor of vacuolar H^+^ ATPase (V-ATPase) that is commonly used to disrupt autophagic degradation. Co-treatment of these two compounds did not induce a further increase in LC3-I/II accumulation, which is indicative of reduced autophagosome degradation by LS-1-10 treatment (Figure 2f). A higher dose of LS-1-10 also led to the disruption of autophagic flux (Supplementary Figure S2A). Consequently, we found an accumulation of cytoplasmic vacuolization and electron-dense particles visualized by transmission electron microscopy, suggesting protein aggregation in LS-1-10-treated cells (Figure 2g). These results indicate that LS-1-10 can inhibit autophagic degradation.

### LS-1-10 blocks autophagosome–lysosome fusion and suppresses lysosomal activity

**Figure 3:**
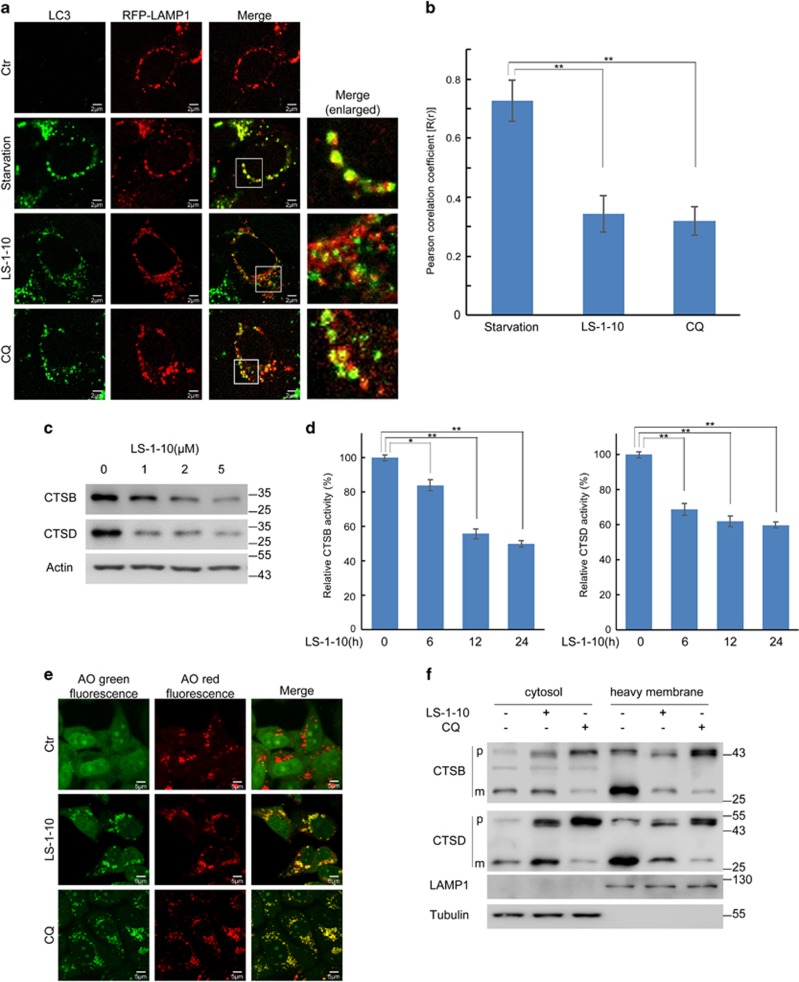
LS-1-10 blocks autophagosome–lysosome fusion and suppresses lysosomal activity. (**a**) DLD1 cells were transfected with RFP-LAMP1, and then treated with 5 *μ*M LS-1-10 or 10 *μ*M chloroquine for 24 h. Co-localization of LC3 with LAMP1 was examined by confocal microscopy. Enlarged images are cropped sections from the merge panels (white line borders), which show the co-localization of the two signals. Scale bars, 2 *μ*m. (**b**) Quantification of the Pearson correlation coefficient to quantify the degree of co-localization. More than 30 cells were counted. Data represent the means±SD (*n*=3). Student’s *t*-test, **P<0.01. (**c**) DLD1 cells were treated with different concentrations of LS-1-10 (1, 2 and 5 *μ*M) for 24 h. Immunoblotting was performed to detect endogenous cathepsin B (CTSB) and cathepsin D (CTSD). (**d**) The enzymatic activities of CTSB and CTSD were analyzed using fluorogenic kits. Data represent the means±S.D. (*n*=3). Student’s *t*-test, *P<0.05; **P<0.01. (e) DLD1 cells treated with 5 *μ*M LS-1-10 or 10 *μ*M chloroquine were subjected to acridine orange (AO) staining. Green fluorescence was acquired at excitation 488 nm and emission 520–560 nm. The red fluorescence was acquired at excitation 488 nm, emission 620 nm by confocal microscopy. (**f**) DLD1 cells were treated with 5 *μ*M LS-1-10 or 10 *μ*M chloroquine for 24 h. Cytoplasmic and heavy membrane proteins were extracted, and endogenous CTSB and CTSD were analyzed by immunoblotting. Abbreviations: p, precursor; m, mature form of CTSB and CTSD

Impaired autophagic degradation is usually a result of inhibited autophagosome–lysosome fusion.^38^ To address whether LS-1-10 affects autophagosome–lysosome fusion, we examined the co-localization of LC3 with LAMP1, which is a lysosome membrane marker. DLD1 cells were transfected with an RFP-LAMP1 plasmid and then subjected to starvation, or LS-1-10 or CQ treatment (whereby starvation served as a positive control and CQ as a negative control). Starvation induced a marked increase in the number of LC3 puncta that co-localized with LAMP1, suggesting normal autophagosome–lysosome fusion to form autolysosomes upon autophagy activation (Figure 3a). By constrast, LS-1-10 prevented autophagosome–lysosome fusion, which mirrored the effect of CQ treatment (Figures 3a and b).

Lysosomal enzymes are functional in an acidic environment.^39, 40^ CQ inhibits autophagy as it raises the lysosomal pH and induces lysosomal membrane permeabilization (LMP).^39, 40^ We therefore examined whether LS-1-10 also downregulates lysosome activity. We examined two proteases – cathepsin B (CTSB) and cathepsin D (CTSD).^41^ Cathepsins are synthesized as inactive precursors in the cytosol and are cleaved to generate an active form within lysosomes.^41^ We found that the active forms of both CTSB and CTSD decreased upon LS-1-10 treatment (Figure 3c). We then measured the enzymatic activities of CTSB and CTSD. Both CTSB and CTSD activities were reduced in a time-dependent manner after LS-1-10 treatment (Figure 3d).

Acridine Orange (AO) is a lysosomotropic metachromatic fluorochrome.^42^ AO emits red fluorescence at high concentrations (when it is present in lysosomes) and green fluorescence at lower concentrations (when it is present in the cytosol and the nucleus; Figure 3e). AO-loaded cells manifested reduced red fluorescence and increased green fluorescence after LMP.^42^ Healthy lysosomes in control cells were stained red whereas lysosomes exposed to LS-1-10 or CQ were predominantly stained yellow (resulting from increased green fluorescence; Figure 3e). These data indicate an increase in lysosomal pH. The translocation of soluble lysosomal components from the lysosomal lumen to the cytosol is a distinctive feature of LMP. We thus monitored the translocation of lysosomal cathepsins by immunoblotting for CTSB and CTSD in various subcellular fractions (cytosol *versus* heavy membranes which includes mitochondria and lysosomes). Here, we observed marked reductions of both CTSB and CTSD in the lysosomes (Figure 3f). Collectively, these data demonstrate that LS-1-10 inhibits autophagosome–lysosome fusion and disrupts lysosomal function.

### LS-1-10 is cytotoxic to colon cancer cells

**Figure 4:**
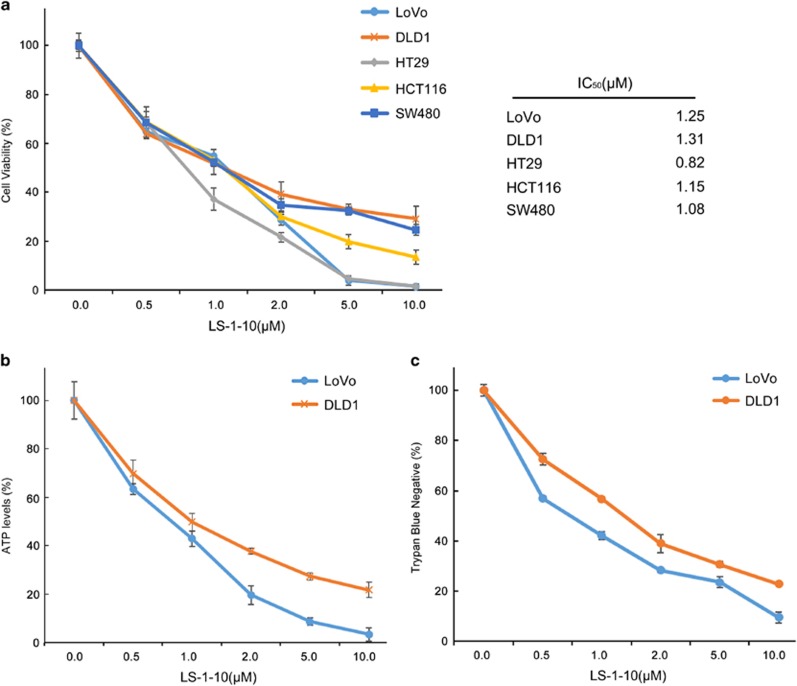
LS-1-10 efficiently reduces the viability of colon cancer cells. (**a**) Five colon cancer cell lines were treated with the indicated concentrations of LS-1-10 for 72 h. Cell viability was measured by CCK-8 assay (left panel). Data represent the means±S.D. (*n*=3). The IC_50_ values were calculated from the results of the CCK-8 assay (right panel). (**b**) DLD1 and LoVo cells were treated with LS-1-10 at the indicated concentrations for 72 h, and ATP levels were measured by ATPlite assay. Data represent the means±S.D. (*n*=3). (**c**) Cells were treated with LS-1-10 for 72 h, and the viability was determined by trypan blue exclusion assay. Data represent the means±S.D. (*n*=3)

Acridine has been used as anticancer drug since 1961 and some of its derivatives can also induce death of cancer cells.^25^ Thus, we investigated the anticancer activity of LS-1-10 by CCK-8 cell viability assay. LS-1-10 reduced cell viability to a similar extent in five colon cancer cell lines (Figure 4a). Comparable results were obtained by ATP assay (Figure 4b) and trypan blue staining (Figure 4c) in two representative cell lines (DLD1 and LoVo).

### LS-1-10 induces DNA damage and caspase 8-mediated apoptosis

**Figure 5:**
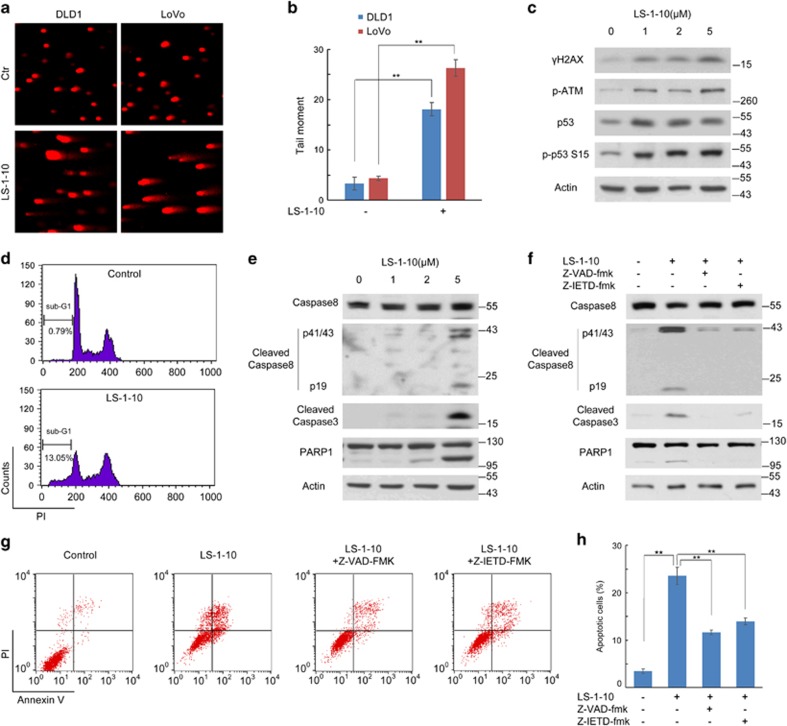
LS-1-10 induces DNA damage and apoptosis. (**a**) DLD1 and LoVo cells were treated with 5 *μ*M LS-1-10 for 48 h, and collected for comet assays. (**b**) Quantification of the data shown in (**a**). Data represent the means±S.D. (*n*=3). Student’s *t*-test, **P<0.01. (**c**) DLD1 cells were treated with different concentrations of LS-1-10 (1, 2, 5 *μ*M) for 48 h. Immunoblotting was performed to detect the phosphorylation of H2AX (Ser129), ATM (Ser1981) and p53 (Ser15). (**d**) DLD1 cells treated with DMSO or 5 *μ*M LS-1-10 were collected for propidium iodide (PI) staining. The DNA contents were analyzed by flow cytometry. (**e**) DLD1 cells were treated with different concentrations of LS-1-10 (1, 2 and 5 *μ*M) for 48 h. Immunoblotting was performed to detect the cleavage of caspase 8, caspase 3 and PARP1. (**f**) DLD1 cells were treated with 5 *μ*M LS-1-10 in the absence or presence of Z-VAD-FMK or Z-IETD-FMK for 48 h. Immunoblotting was performed to detect the cleavage of caspase 8, caspase 3 and PARP1. (**g**) Cells treated as in (**f**) were subjected to flow cytometry to detect AnnexinV/PI staining. (**h**) Quantification of the apoptotic cells shown in (**d**). Data represent the means±S.D. (*n*=3). Student’s *t*-test, **P<0.01

As LS-1-10 exhibits efficient anticancer activity, we focused our attention on the potential mechanism underlying its cytotoxicity in cancer cells. The linear tricyclic aromatic structure of acridine permits DNA intercalation.^43^ Acridine compounds, such as m-AMSA and DACA, have also been reported to be topoisomerase I/II inhibitors and cause DNA double-strand breaks (DSBs).^44^ Therefore, we used a comet assay to determine whether LS-1-10 also induces DNA damage. Cells were treated with 5 *μ*M LS-1-10 for 24 h and then harvested. The LS-1-10-treated cells exhibited a greater DNA tail area and longer DNA tail length than control cells, indicating more extensive DNA damage (Figure 5a). The degree of DNA damage was analyzed using CometScore (Sumerduck, VA, USA) software (Figure 5b) and the data suggested that LS-1-10 causes marked DNA damage in multiple colon cancer cells.

We next investigated whether LS-1-10-induced DNA damage was achieved by generating DSBs. We detected H2AX phosphorylation (*γ*-H2AX), a hallmark of DSBs and found that *γ*-H2AX increased in LS-1-10-treated cells in a dose-dependent manner compared to untreated cells (Figure 5c). We also detected activation of ATM and phosphorylation of its downstream target p53 (Figure 5c). Immunostaining for DLD1 also indicated *γ*-H2AX foci formation upon treatment with LS-1-10 (Supplementary Figure S3A and S3B). These results support that LS-1-10 induces DSBs in cancer cells.

DNA damaging agents cause cell cycle arrest and apoptosis in response to stress.^45^ To determine whether LS-1-10 elicits cell cycle arrest, we collected LS-1-10-treated cells and performed flow cytometry analysis by propidium iodide (PI) staining. As expected, there was notable S/G2 arrest in cells after LS-1-10 treatment (Figure 5d). We also detected an increase in the fraction of the cells with DNA content less than G0/G1 cells (region of sub-G1 in Figure 5d), which are commonly considered to be apoptotic cells. We next evaluated the cleavage of typical markers of apoptosis, namely caspase 9, caspase 8, caspase 3 and PARP1. Cleavage of caspase 8 (rather than caspase 9) and its downstream caspase 3 and PARP1 increased in a dose-dependent manner upon LS-1-10 treatment (Figure 5e and Supplementary Figure S3C). We further confirmed that LS-1-10 induces caspase 8-mediated apoptosis using a pan-caspase inhibitor (Z-VAD-FMK) and a caspase 8-specific inhibitor (Z-IETD-FMK). Both Z-VAD-FMK and Z-IETD-FMK effectively decreased the cleavage of caspase 3 and PARP1 (Figure 5f). This finding was confirmed by flow cytometric analysis of AnnexinV/PI stained cells, which showed that Z-VAD-FMK and Z-IETD-FMK blocked LS-1-10-induced apoptosis (Figures 5g and h). Collectively, these data support that LS-1-10 is a DNA-damaging agent and induces caspase 8-mediated apoptosis.

### LS-1-10 possesses more efficient anticancer activity than other conventional chemotherapeutic agents

**Figure 6:**
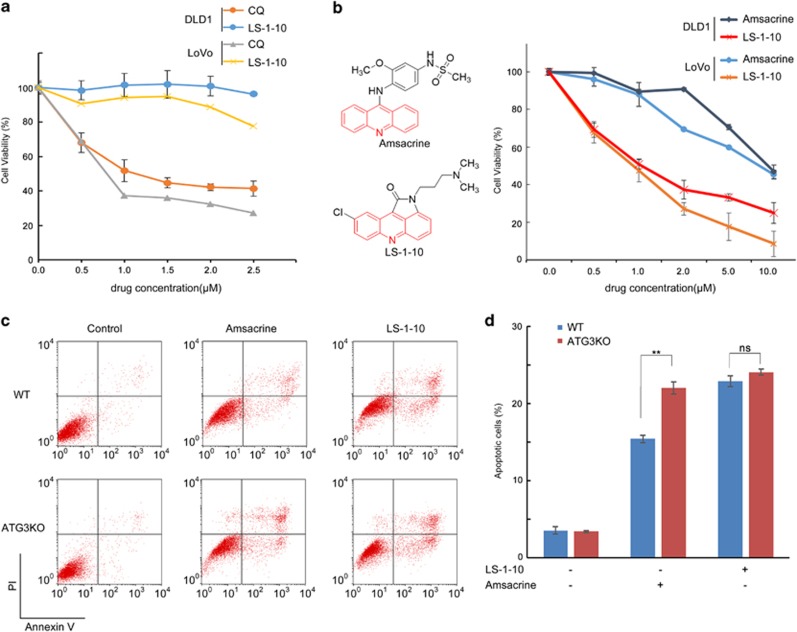
LS-1-10 reduces cancer cell viability. (**a**) DLD1 and LoVo cells were treated with varying concentrations of LS-1-10 or chloroquine (CQ) as indicated for 72 h. Cell viability was measured by CCK-8 assay. Data represent the means±S.D. (*n*=3). (**b**) DLD1 and LoVo cells were treated with LS-1-10 or amsacrine (chemical structures shown in the left panel) at the indicated concentrations for 72 h. Cell viability was measured by CCK-8 assay. Data represent the means±S.D. (*n*=3). (**c**) DLD-1 cells were treated with 5 *μ*M LS-1-10 or 5 *μ*M amsacrine for 48 h, and then subjected to flow cytometry to detect AnnexinV/propidium iodide staining. (**d**) Quantification of the apoptotic cells shown in (**c**). Data represent the means±S.D. (*n*=3). Student’s *t*-test, **P<0.01

To further characterize the efficacy of LS-1-10, we compared the anticancer activity of LS-1-10 with CQ. LS-1-10 was significantly more potent at reducing cell viability than CQ (>50%), despite similar autophagy-inhibiting potency (Figure 6a).

As previously discussed, m-AMSA is a chemotherapeutic used to treat lymphoma and leukemia.^44^ Similar to LS-1-10, m-AMSA is an acridine derivative (Figure 6b) and induces a high level of autophagy in cancer cells.^31^ We hypothesized that LS-1-10 might be more efficient at reducing the viability of cancer cells than m-AMSA as it blocks cytoprotective autophagy. Consistently, LS-1-10 was more potent than m-AMSA at reducing colon cancer cell viability (Figure 6b), as determined by CCK-8 assay. To further characterize the role of autophagy in cell death, we treated wild-type (WT) and autophagy-deficient ATG3KO cells with either m-AMSA or LS-1-10. Flow cytometric analysis of AnnexinV/PI stained cells found that compared to WT cells, ATG3KO cells had increased sensitivity to m-AMSA but not LS-1-10 (Figures 6c and d). This finding was consistent with the autophagic flux in these cells.

Lucanthone is an autophagy inhibitor that is more potent than CQ at reducing breast cancer cell viability.^46^ Lucanthone is also able to disrupt topoisomerase II activity and inhibit APE1 – an important enzyme involved in DNA base excision repair.^47, 48^ Therefore, we assessed whether LS-1-10 is more efficient than lucanthone in reducing cancer cell viability. LS-1-10 was more effective than lucanthone at reducing the viability of multiple cancer cells (Supplementary Figure S5). Collectively, these data indicate that LS-1-10, as a single-agent, has higher anticancer efficacy than other similar drugs.

### The anticancer and autophagy-inhibiting activities of LS-1-10 *in vivo*

**Figure 7:**
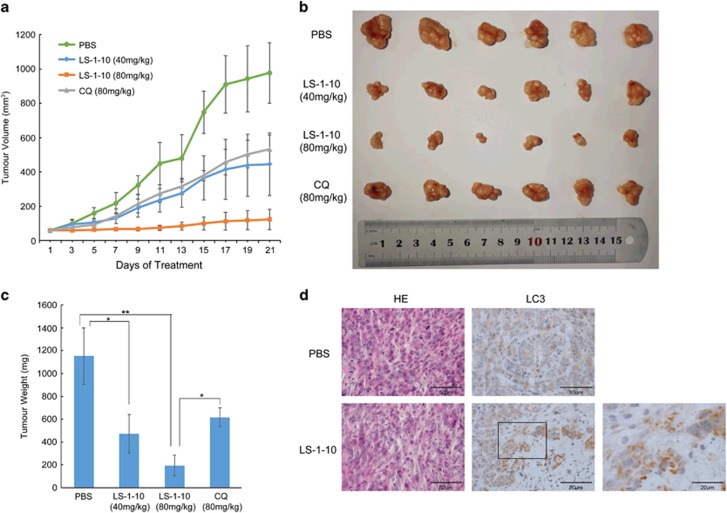
LS-1-10 exhibits anticancer and autophagy-inhibiting activities *in vivo*. (**a**) Four-week-old nude mice were engrafted with DLD1 cells and randomly divided into four groups (*n*=6). After 2 weeks, the tumor-bearing mice were treated with PBS, LS-1-10 40 mg/kg, LS-1-10 80 mg/kg, or CQ 80 mg/kg by intraperitoneal injection every 5 per7 (5/7) days for 3 weeks. Tumor volumes were calculated by measuring the length and width using Vernier calipers every 2 days. Data represent the means±S.D. (*n*=6). (**b**) Images of the tumors from **a**. (**c**) Quantification of the tumor weights from (**a**). Student’s *t*-test, *P<0.05; **P<0.01. (**d**) Immunohistochemical staining of LC3 in tumor sections treated with PBS or LS-1-10. Four-week-old nude mice were engrafted with DLD1 cells and observed until tumors reached ~100mm^3^. Tumor-bearing mice were then treated with PBS or 20 *μ*g LS-1-10 by intra-tumoral injection once every 2 days for a total of seven injections. Mice were killed and tumors were resected 2 days after the final injection

The fumarate salt of LS-1-10 was synthesized to enhance its aqueous solubility for use in *in vivo* studies (‘LS-1-10’ refers to the fumarate of LS-1-10 in all the *in vivo* experiments). To determine the anticancer activity of LS-1-10 *in vivo*, nude mice were injected with DLD1 cells and 2 weeks later were treated with PBS, LS-1-10 40 mg/kg LS-1-10 80 mg/kg or CQ 80 mg/kg by intraperitoneal injection every 5 per 7 (5/7) days for 3 weeks. The tumor sizes and weights were significantly lower in the two LS-1-10-treated groups (Figures 7a–c). LS-1-10 was also more efficient at reducing the tumor sizes and weights when compared to the same dose of CQ treatment (Figures 7a–c). Immunohistochemical analysis of LC3 distribution found that LC3 was diffuse in the cytoplasm of PBS-treated mice, but was present in the large number of punctuates in tumor tissues of LS-1-10-treated mice (Figure 7d). These data support that LS-1-10 inhibits autophagic degradation *in vivo* and confirm the anticancer and autophagy-inhibiting activities of LS-1-10.

## Discussion

This study developed a novel derivative of acridine, LS-1-10, with a similar structure to CQ and with autophagy-inhibiting activity. LS-1-10 suppresses autophagy-mediated degradation by inhibiting autophagosome–lysosome fusion and disrupting lysosome function. This molecule with autophagy-inhibiting and DNA-damaging activity reduced the viability of numerous tested colon cancer cell lines.

LS-1-10 shares the same acridine backbone as m-AMSA, which was the first synthetic DNA-intercalating agent successfully used in a clinical setting (Figure 6b).^49, 50^ Both LS-1-10 and m-AMSA possess the DNA-binding domain acridine moiety and the N-appendage moiety that permits topoisomerase II binding (Figure 6b). m-AMSA is an effective treatment for acute leukemia and malignant lymphoma, but is ineffective against solid tumors.^49, 50^ The tumor microenvironment has a crucial role in mediating the chemoresistance of tumor cells.^51, 52, 53^ Solid tumors typically exhibit a hypoxic environment, which results in increased metabolic stress and thus induces autophagy.^52^ Previous studies have verified that m-AMSA-induced autophagy has a cytoprotective role.^31^ Consequently, we speculate that the decreased sensitivity of solid tumors to m-AMSA may be due to high basal levels of autophagy. As such, the effect of LS-1-10 on inhibiting autophagy may be a promising therapeutic approach for solid tumors. As expected, LS-1-10 was more effective than m-AMSA at reducing the viability of colon cancer cells (Figure 6b).

High levels of autophagy are induced in response to most DNA-damaging cancer therapeutics such as etoposide, m-AMSA or irradiation, to generate energy, maintain homeostasis and promote survival of cancer cells.^54^ Even though autophagy-mediated cell death has been reported in cancer cells, a high-content screen of ~1400 cytotoxic agents found that no single compound could induce cell death by autophagy, underscoring that autophagy is predominantly cytoprotective in cancer cells in response to anti-neoplastic therapies.^31^ Our colony formation analysis confirmed this cytoprotective function of autophagy in colon cancer cell lines (Supplementary Figures S6A and B). Specifically, Cas9 knockout of ATG3/ATG7, could disrupt autophagy flux and sensitize the cancer cells to etoposide (Supplementary Figures S6A and B). Disruption of autophagic degradation may, therefore, augment the efficacy of therapeutics used in colon cancer.

DNA damage-induced apoptosis is typically mediated by caspase 9, rather than caspase 8.^45^ Interestingly, we found that LS-1-10 triggered caspase 8-mediated DNA damage-induced apoptosis (Figure 5). It remains to be confirmed what causes caspase 8 cleavage here. Previous studies reported that cytosolic CTSD primes caspase 8 (but not caspase 9) activation by proteolysis.^55^ Consistently, we found that LS-1-10 treatment induces LMP and triggers CTSD diffusion throughout the cytosol (Figures 3e and f). We previously hypothesized that disruption of lysosomal function and the subsequent diffusion of cathepsins might be the main cause of caspase 8 activation and apoptosis. Agents that induce LMP trigger apoptosis independent of functional p53 status.^56^ In support of our hypothesis, we found the anticancer activity of LS-1-10 to be independent of p53 (Supplementary Figure S4). However, knockdown of CTSD only partly rescued the cleavage of caspase 8 and its substrates (Supplementary Figure S3D). Thus, we propose it is likely that other pathways are also involved in the regulation of caspase 8 activation and apoptosis and that lysosomal dysfunction may not be the main trigger of apoptosis.

Although the effects of LS-1-10 on inhibiting autophagy and inducing apoptosis are driven by different molecular mechanisms, we consider that the autophagy-inhibiting activity of LS-1-10 could augment its anticancer efficacy. Both key DNA damage related proteins, such as Sae2 (human CtIP)^57^, p62^54^ and active caspase 8 can be degraded via autophagic pathways.^58^ These two aspects contribute to the cytotoxicity of LS-1-10. The specific targets of LS-1-10 in the lysosome have not been identified, thus our hypothesis and the precise contribution of autophagy inhibition to the cytotoxicity of LS-1-10 remains to be confirmed. Our cell viability analysis of LS-1-10 analogs provides some indirect support as those compounds that showed no autophagy-inhibiting activity were also less effective at reducing the viability of DLD1 cells than LS-1-10 (Supplementary Figure S5C).

CQ, HCQ and lucanthone are the only clinically relevant autophagic inhibitors currently in use as cancer therapeutics.^59^ However, the ocular toxicity of CQ and HCQ has severely limited their utilization. Novel inhibitors of autophagy, including many analogs of CQ, have been identified by different groups.^60^ Even though they may have lower toxicity and a better therapeutic index, unexpected adverse effects due to drug–drug interactions may still occur. The development of a single-agent autophagy inhibitor with anticancer activity will likely be the most effective and safe strategy. Lys05 is an autophagy inhibitor that exhibits single-agent anticancer activity without inducing toxic effects in mice.^61^ Lys05 is a dimeric form of CQ but exerts10-fold higher autophagy inhibition compared to CQ.^61^ The Lys05 design strategy encouraged us to synthesize and test the dimeric analog of LS-1-10. Preliminary data showed that the IC_50_ of dimeric LS-1-10 is <10 nM (unpublished data). Further investigations are now needed to clarify its biological activity.

The anti-schistome agent lucanthone shares the same autophagy-inhibiting and DNA-damaging activities as LS-1-10.^46^ However, two phase II clinical trials of lucanthone were prematurely terminated or suspended for unknown reasons. Our analysis found that LS-1-10 has higher efficacy than lucanthone at reducing the viability of multiple colon cancer cells (Supplementary Figures S5A and B). This effect may be due to the DNA-intercalating activity of LS-1-10 as lucanthone does not have a DNA-binding domain.^47, 48^

In summary, our study describes the first acridine derivative with autophagy-inhibiting activity in cultured colon cancer cell lines. LS-1-10 has potent *in vitro* anticancer activity achieved by inducing DNA damage and caspase 8-mediated apoptosis. The autophagy-inhibiting activity of LS-1-10 may augment its efficacy at reducing the viability of cancer cells. Our results demonstrate that LS-1-10 is a potent anticancer agent that may be a valuable cancer therapeutic in the future.

## Materials and methods

### Reagents

All acridine derivatives, including LS-1-10 were synthesized as previously described.^62^ CQ (C6628) and etoposide (E1383) were purchased from Sigma-Aldrich (St. Louis, MO, USA); bafilomycin A1 (S1413) was purchased from Selleck Chemicals (Houston, TX, USA); Z-VAD-fmk (KGA8254) and Z-IETD-fmk (KGA8260) were purchased from Keygen Biotech (Nanjing, China); Amsacrine (T1206) was purchased from TargetMol (MA, USA); Lucanthone (L473700) was purchased from Toronto Research Chemicals Inc (Toronto, Ontario, Canada).

### Antibodies

MAP1LC3 (2775), PARP1 (9542), *γ*H2AX (9718), cleaved caspase8 (9496), caspase3 (9662), phospho-ATM (Ser1981) (5883) and phosphor-p53 (Ser15) (9284) were purchased from Cell Signaling Technology (Danvers, MA, USA); Atg3 (M133-3), p62/SQSTM1 (PM045) and *α*-tubulin (PM054) were purchased from MBL (Naka-ku, Nagoya, Japan); p53 DO-1 (sc-126) and *β*-Actin (sc-7210) were purchased from Santa Cruz Biotechnology Inc. (Santa Cruz, CA, USA); Cathepsin D (ab75852), LAMP-1 (ab25630) and Caspase 8 (full-length) (ab32397) were purchased from Abcam (Cambridge, MA, USA); Cathepsin B (12216-1-AP) and caspase 9 (66169-1-LG) were purchased from Proteintech Group Inc (Chicago, IL, USA).

### Plasmids

The ptf-LC3 plasmid was purchased from Addgene (Cambridge, MA, USA) (Plasmid #21074).

### Cell culture and transfection

DLD1 and LoVo cell lines were purchased from the American Type Culture Collection, and grown in RPMI or DMEM medium supplemented with 10% fetal bovine serum in a 37 °C incubator with a humidified, 5% CO_2_ atmosphere. HCT116^(+/+)^ and HCT116^(-/-)^ cell lines were grown in DMEM supplemented with 10% fetal bovine serum in a 37 °C incubator with a humidified, 5% CO_2_ atmosphere. Lipofectamine 2000 (Invitrogen, Waltham, MA, USA) was used for transfection, according to the manufacturer’s instructions.

### Generation of Cas9 *ATG3/ATG7* knockout cell line

The Cas9 *ATG3* knockout cell lines were generated by CRISPR–Cas9 method in DLD1 cells using the SpCas9-2 A-Puro vector (Addgene).^63^ The *ATG3/ATG7* sgRNA was designed using online software (http://crispr.mit.edu). The sgRNA *ATG3* sequence was 5′-GTGAAGGCATACCTACCAAC-3′ and the sgRNA *ATG7* sequence was 5‘-AACTCCAATGTTAAGCGAGC-3′. The plasmids were transfected into DLD1 cells and selected in 2.5 *μ*g/ml puromycin.

### Cytosol and heavy membrane fractionation

Cells (at a density of 3x10^7^/ml) were permeabilized with digitonin for 5 min on ice in cytosolic extraction buffer (250 mM sucrose, 70 mM KCl, 137 mM NaCl, 4.3 mM Na_2_HPO_4_, 1.4 mM KH_2_PO_4_ pH 7.2, 200 *μ*g/ml digitonin) supplemented with a complete protease inhibitor cocktail (Roche, Mannheim, Germany). Permeabilization of the plasma membrane was confirmed by 0.2% trypan blue staining. Cells were then centrifuged at 1000 × *g* for 5 min at 4 °C. The supernatant (cytosolic fraction) was saved and the pellet (heavy membrane) was solubilized in the same volume of lysis buffer (50 mM Tris pH 7.4, 150 mM NaCl, 2 mM EDTA, 2 mM EGTA, 0.2% Triton X-100, 0.3% NP-40) supplemented with protease inhibitor cocktail, followed by the precipitation of materials to be discarded at 10 000 × *g* for 10 min at 4 °C.

### Immunoblot assay

In brief, cells were collected using a scraper and washed once with cold PBS. The cells were then lysed in lysis buffer (50 mM Tris-HCl, 250 mM NaCl, 5 mM EDTA, 50 mM NaF, 0.1% NP-40) supplemented with 1% protease inhibitor cocktail. Equal amounts of proteins were size-fractionated by 7.5–15% SDS-PAGE. At least three independent experiments were performed.

### Real-time PCR analysis of mRNA

Total RNA was isolated using TRIzol reagent. The cDNA was synthesized from 2 *μ*g of RNA using a Quantscript RT Kit (TianGen, Beijing, China, KR103). The following primers were used for RT PCR: *p62*, forward: 5′-AGCTGCCTTGTACCCACATC-3′, reverse: 5′-CAGAGAAGCCCATGGACAG-3′ *ACTB*, forward: 5′-CCAACCGCGAGAAGATGA-3′, reverse: 5′-CCAGAGGCGTACAGGGATAG-3′.

### Immunofluorescence

Cells were cultured in confocal dishes to ~60% confluence. After transfection and treatment, cells were fixed with 4% paraformaldehyde and permeabilized with 100% methanol. The dishes were incubated in blocking solution (0.8% BSA in PBS) and exposed overnight to primary antibody (1 : 100 dilution for all antibodies) at 4 °C. The cells were then washed three times with blocking solution and then exposed to a secondary antibody (1 : 100 dilution) conjugated to FITC/TRITC. Cells were observed and analyzed under a confocal microscope (Olympus BX-51, America Inc.).

### CCK-8 assay

Equal numbers of cells (~5,000/well) were seeded into a 96-well plate 24 h before experimentation. Cells were treated with different compounds for 72 h. After treatment, CCK-8 was added to the 96-well plate and incubated at 37 °C for 1 h. The absorbance of each sample was read at 450 nm.

### Comet assay

The comet assay was performed as described previously.^64^ In brief, frosted microscopic slides were covered with 0.5% normal melting agarose at 60 °C. LS-1-10-treated or untreated cells (~10^5^) in PBS were mixed with an equal amount of 1% low-melting agarose to form a cell suspension. After electrophoresis, slides were examined at × 20 magnification and images were acquired under a confocal microscope (Olympus BX-51, America Inc., Center Valley, PA, USA).

### Cathepsin activity assay

The catalytic activities of cathespins were determined using CTSB and CTSD Activity Fluorometric Assay Kits (BioVision (Milpitas, CA, USA), K140-100, K143-100,). Briefly, 10^6^ cells were collected by centrifugation and lysed in 200 *μ*l chilled cell lysis buffer. Then 50 *μ*l cell lysate was transferred to 96-well plates, mixed with reaction buffer and substrate, and incubated at 37 °C for 2 h. The samples were read in a fluorometer with 400 nm excitation and 505 nm emission filters. The activity was normalized to the protein concentration.

### Acridine orange staining

Acridine orange (5 *μ*g/ml; Sigma (St. Louis, MO, USA) BR-A60145) was added to cells for 10 min (37 °C, 5% CO_2_). After three washes with PBS, the cells were observed under a confocal microscope (Olympus BX-51, America Inc.) equipped with an argon laser (excitation wavelength 488 nm), and a 100 × objective lens and images were acquired. Acridine orange produces a red fluorescence (emission filter 620 nm long pass) when within lysosomal compartments, and a green fluorescence (emission between 520 and 560 nm) in cytosolic and nuclear compartments.

### Flow cytometry

Apoptotic cells have a lower DNA content than normal cells and appear as a sub-G1 peak on a DNA cell cycle histogram. Here, cells were harvested to quantify the level of apoptosis after various treatments. In brief, cells were dissociated using trypsin and washed once with cold PBS. Cells were then fixed with 70% ethanol and stored overnight at −20 °C. Propidium Iodide (10 *μ*g/ml; Sigma) was added to stain cells in the presence of RNase at 37 °C for 10 min, and the cells were then analyzed on a FACscan Flow Cytometer (Becton Dickinson, Franklin Lakes, NJ, USA) with manual gating using CellQuest software (Franklin Lakes, NJ, USA).

### Tumorigenesis in nude mice

Four-week-old BALB/c nude mice were purchased from the Experimental Animal Centre of Peking University Health Science Centre (Beijing, China) and housed in a pathogen-free environment. DLD1 cells (~2 × 10^6^) were delivered into animals via hypodermic injection. After 2 weeks, all mice were randomly divided into four groups (*n*=6) and administered PBS, LS-1-10 40 mg/kg, LS-1-10 80 mg/kg, or CQ 80 mg/kg via intraperitoneal injection every 5–7 days. At the end of the experiment (5 weeks after tumor implantation), the mice were sacrificed and the weight of each tumor was determined.

### Statistical analysis

Three independent experiments were performed prior to statistical analysis. The data represent the means±S.D. A *P*<0.05, by unpaired Student’s *t*-test, was considered statistically significant.
