# Supplementary figures and images for: A novel acridine derivative, LS-1-10 inhibits autophagic degradation and triggers apoptosis in colon cancer cells
Source: Cell Death Dis. 2017 Oct 5;8(10):e3086–. doi: 10.1038/cddis.2017.498 (PMC5682664; doi:10.1038/cddis.2017.498)

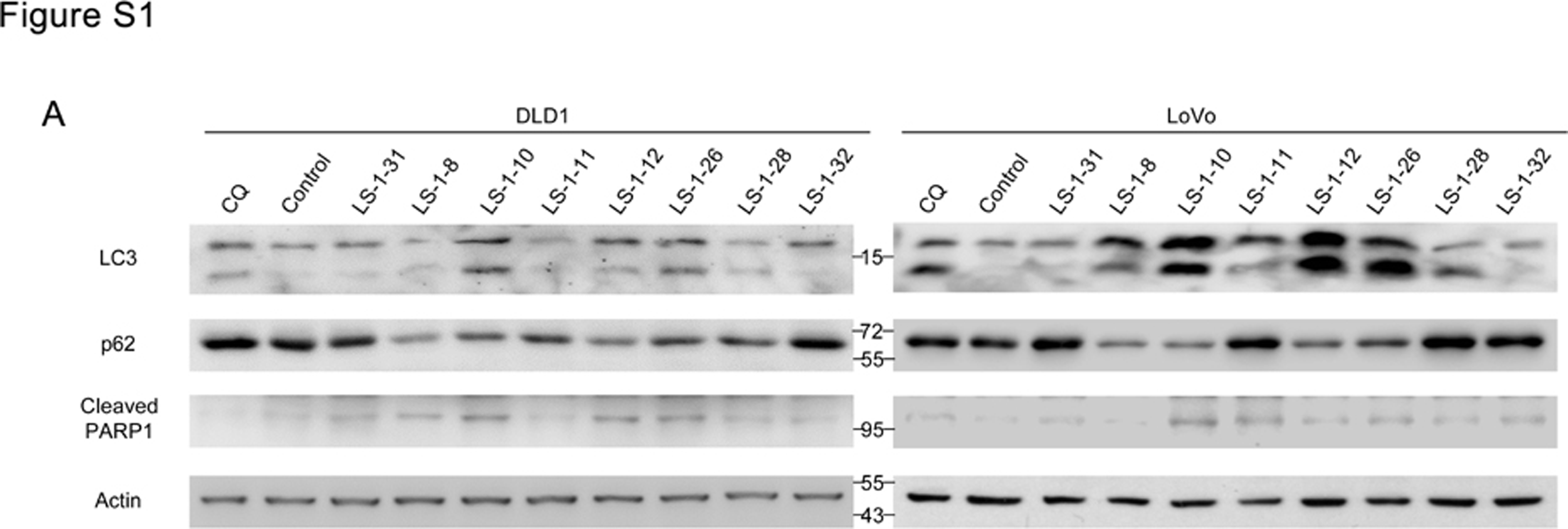

Supplement: Supplementary Figure S1 [file cddis2017498x1.tif]

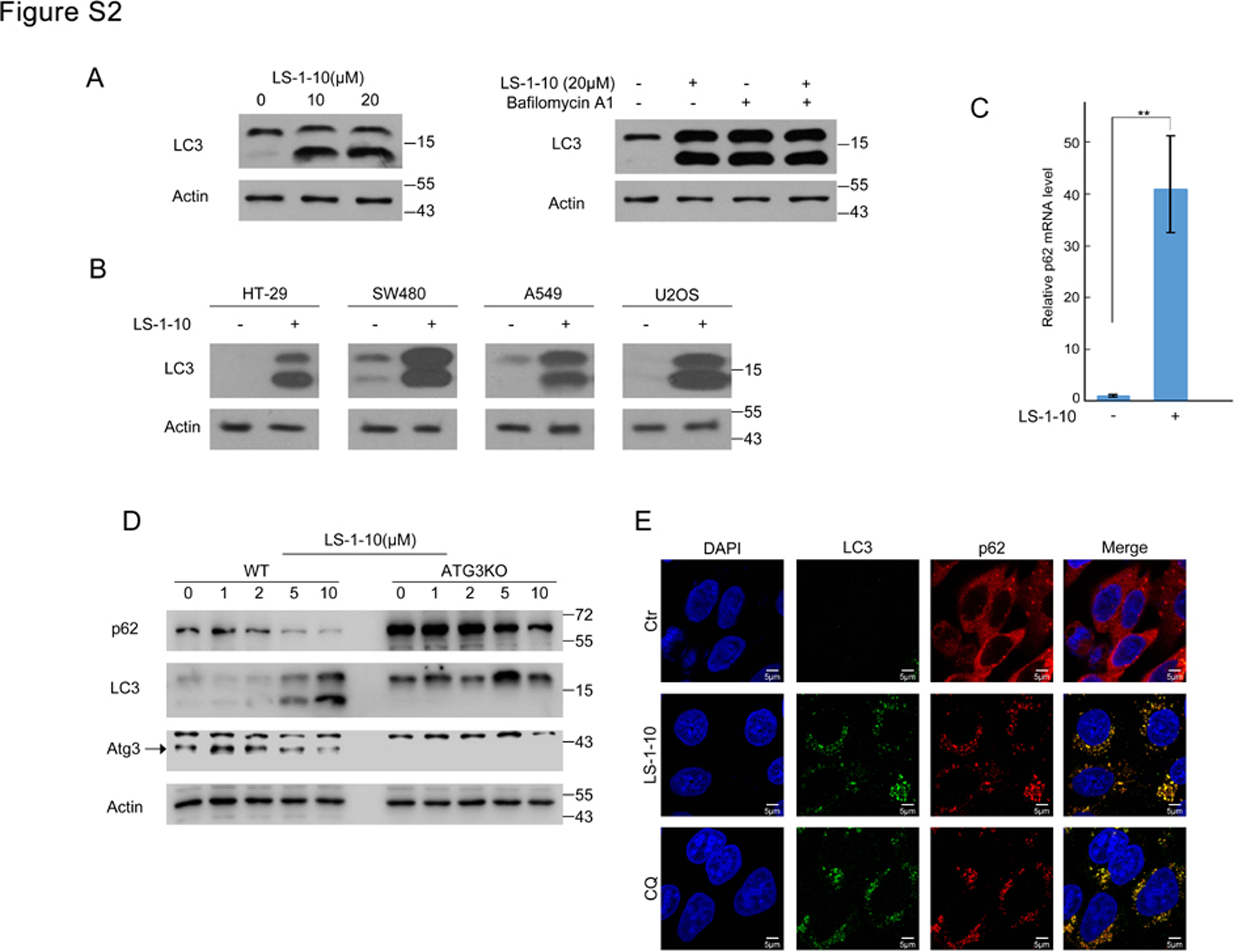

Supplement: Supplementary Figure S2 [file cddis2017498x2.tif]

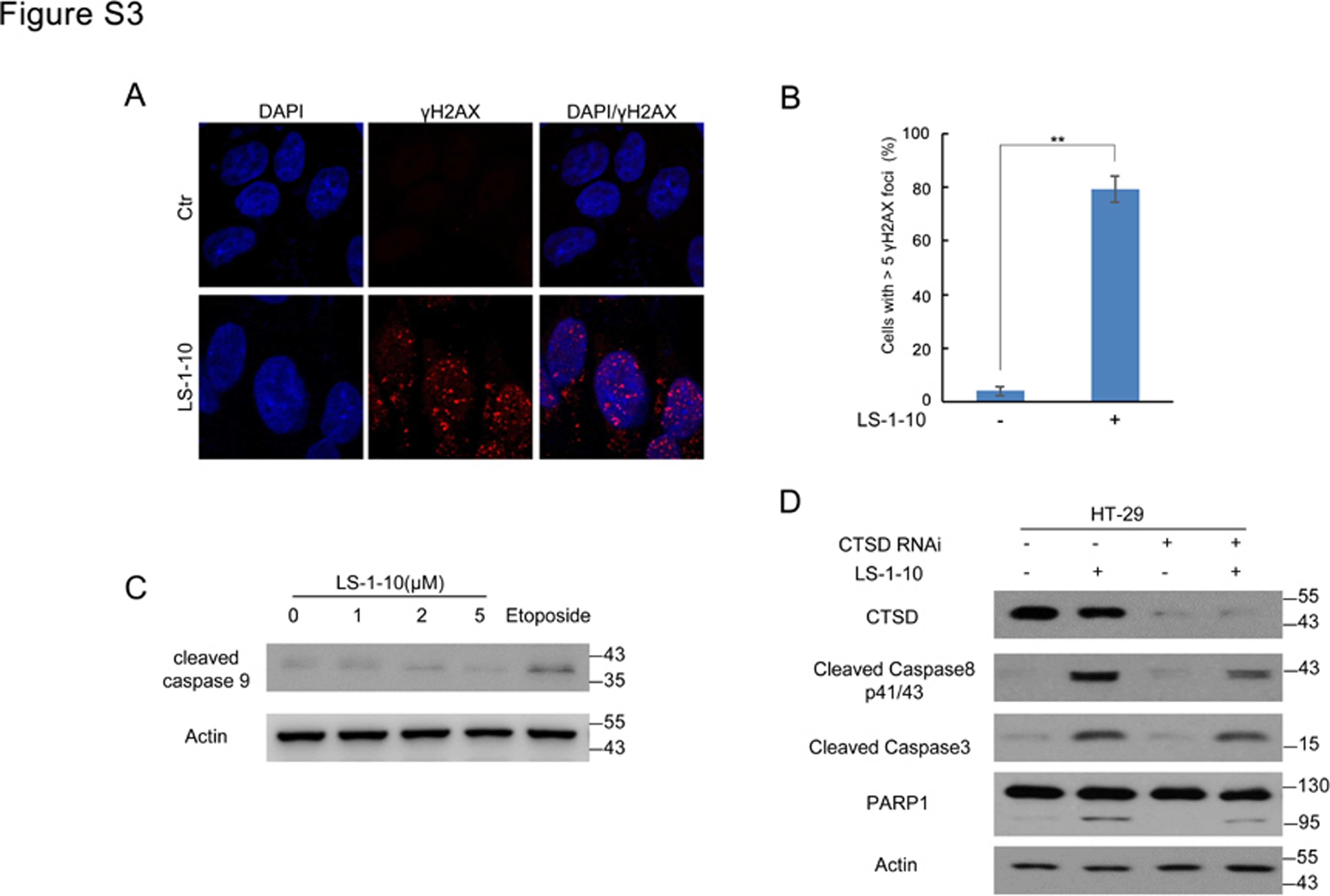

Supplement: Supplementary Figure S3 [file cddis2017498x3.tif]

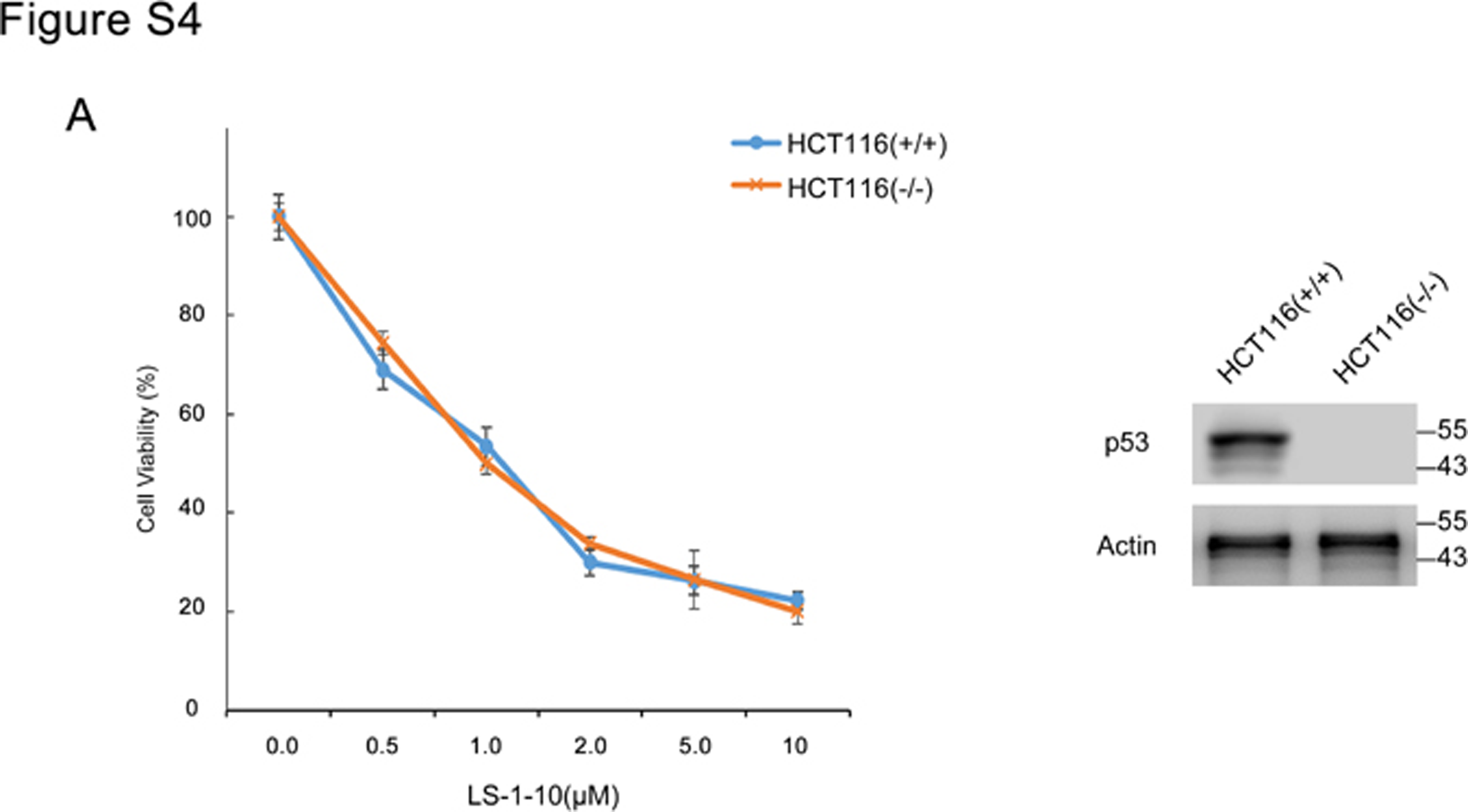

Supplement: Supplementary Figure S4 [file cddis2017498x4.tif]

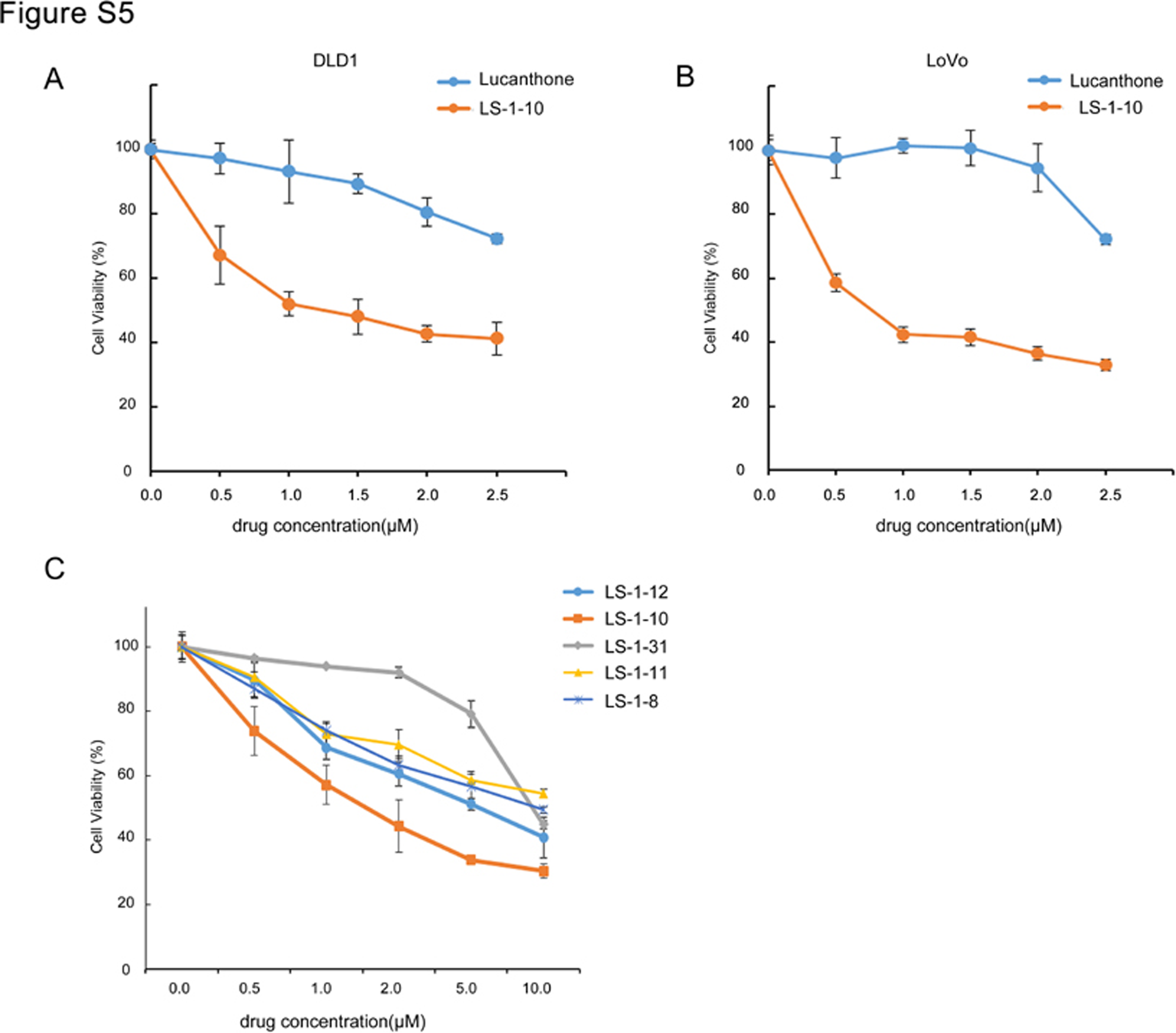

Supplement: Supplementary Figure S5 [file cddis2017498x5.tif]

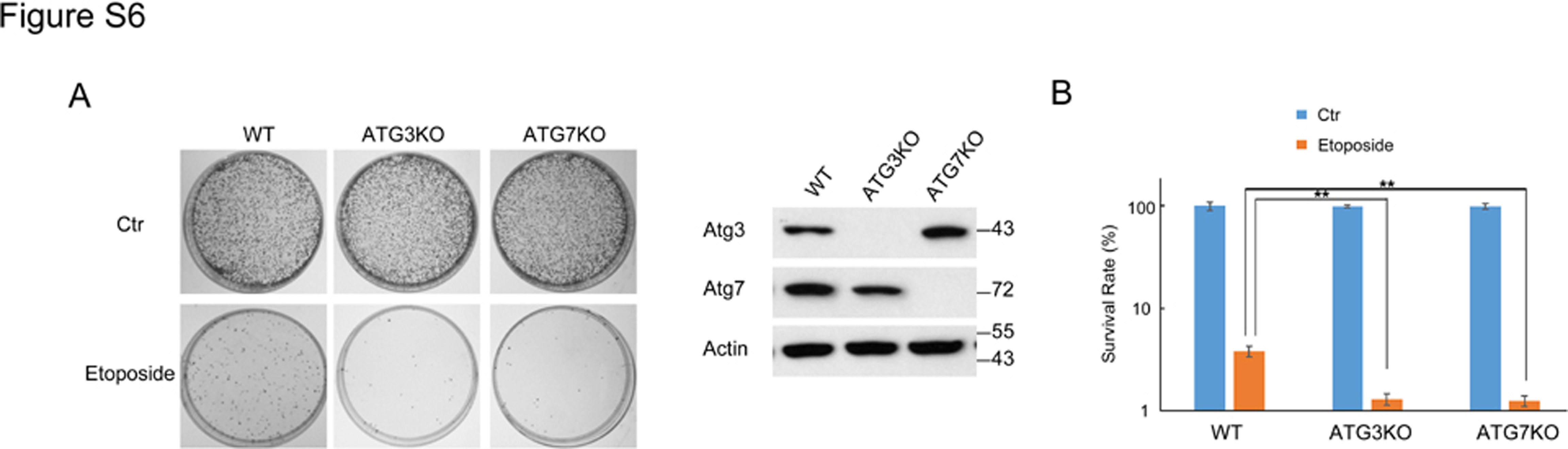

Supplement: Supplementary Figure S6 [file cddis2017498x6.tif]
