# Supplementary material for: A novel acridine derivative, LS-1-10 inhibits autophagic degradation and triggers apoptosis in colon cancer cells
Source: Cell Death Dis. 2017 Oct 5;8(10):e3086–. doi: 10.1038/cddis.2017.498 (PMC5682664; doi:10.1038/cddis.2017.498)
Supplement: Supplementary Figure Legends [file cddis2017498x7.doc]

**Supplemental Figure Legends**

**Supplemental Figure S1.** DLD1 cells were treated with 5 μM acridine derivatives at or 10 μM chloroquine for 24 h. Immunoblotting was performed to detect endogenous LC3, p62/SQSTM1 and cleaved PARP1.

**Supplemental Figure S2.** (A) DLD1 cells were treated with different concentrations of LS-1-10 (10, 20 µM) for 24 h. Immunoblotting was performed to detect endogenous LC3 (left panel). DLD1 cells were treated with 20 μM LS-1-10, 100 nM bafilomycin A1, or both agents for 24 h. Whole-cell lysates were extracted for immunoblotting with the indicated antibodies (right panel). (B) HT-29, SW480, A549 and U2OS cells were treated with 5 μM LS-1-10 for 24 h. Immunoblotting was performed to detect endogenous LC3. (C) Relative p62 mRNA expression (compared with actin) in DLD1 cells treated with LS-1-10, as determined by quantitative RT-PCR. Data represent the means ± SD (n=3). Student’s *t*-test, ***P*< 0.01. (D) Wild type (WT) or ATG3 knock-out (KO) DLD1 cells were exposed to different concentrations of LS-1-10 (1, 2, 5, 10 µM) for 24 h. Immunoblotting was performed to detect endogenous p62, LC3 and Atg3. (E) DLD1 cells were incubated with 5 μM LS-1-10 or 10 μM chloroquine (CQ) for 24 h. Immunofluorescence was performed after staining with LC3/p62 antibodies. Scale bars, 5 µm.

**Supplemental Figure S3.** (A) DLD1 cells were treated with 5 μM LS-1-10 for 24 h. Immunofluorescence was performed after staining with an γH2AX antibody. Scale bars, 5 µm. (B) Quantification of cells with γH2AX foci shown in (D). Cells with more than five foci in the nucleus were counted. Data represent the means ± SD (n=3). Student’s *t*-test, ***P*< 0.01. (C) DLD1 cells were treated with different concentrations of LS-1-10 (1, 2, 5 µM) or etoposide (10 µM) for 48 h. Immunoblotting was performed to detect cleavage of caspase 9. (D) DLD1 cells were transfected with cathepsin D (CTSD) siRNA, and then treated with 5 μM LS-1-10 for 24 h. Whole-cell lysates were extracted for immunoblotting with the indicated antibodies.

**Supplemental Figure S4.** HCT116^(+/+)^ and HCT116^(-/-)^ cells were treated with different concentrations of LS-1-10 as indicated, for 72 h. Cell viability was measured by CCK-8 assay. Data represent the means ± SD (n=3) (left panel). Immunoblotting was performed to detect endogenous p53 (right panel).

**Supplemental Figure S5.** (A) DLD1 and (B) LoVo cells were treated with different concentrations of LS-1-10 or lucanthone as indicated, for 72 h. Cell viability was measured by CCK-8 assay. Data represent the means ± SD (n=3). (C) DLD1 cells were treated with different concentrations of LS-1-8, LS-1-10, LS-1-11, LS-1-12, LS-1-31 as indicated, for 72 h. Cell viability was measured by CCK-8 assay. Data represent the means ± SD (n=3).

**Supplemental Figure S6.** (A) Wild-type (WT) or autophagy-deficient DLD1 cells were treated with DMSO or 20 μM etoposide for 2 h. After 2 weeks, colony formation was analyzed by crystal violet staining (left). Immunoblotting was performed to detect endogenous ATG3 and ATG7 expression (right). (B) Statistical analysis of the surviving fraction in (A). Data represent the means ± SD (n=3). ***P*< 0.01.
